# Long-Term Outcome and Role of Biology within Risk-Adapted Treatment Strategies: The Austrian Neuroblastoma Trial A-NB94

**DOI:** 10.3390/cancers13030572

**Published:** 2021-02-02

**Authors:** Stefan Fiedler, Inge M. Ambros, Evgenia Glogova, Martin Benesch, Christian Urban, Marlene Mayer, Georg Ebetsberger-Dachs, Edit Bardi, Neil Jones, Agnes Gamper, Bernhard Meister, Roman Crazzolara, Gabriele Amann, Karin Dieckmann, Ernst Horcher, Reinhold Kerbl, Bettina Brunner-Herglotz, Andrea Ziegler, Peter F. Ambros, Ruth Ladenstein

**Affiliations:** 1 St. Anna Children’s Hospital, 1090 Vienna, Austria; stefan.fiedler@stanna.at (S.F.); edit.bardi@keplerklinikum.at (E.B.); 2 CCRI, Children’s Cancer Research Institute, 1090 Vienna, Austria; inge.ambros@ccri.at (I.M.A.); evgenia.glogova@ccri.at (E.G.); bettina.brunner-herglotz@ccri.at (B.B.-H.); andrea.ziegler@ccri.at (A.Z.); peter.ambros@ccri.at (P.F.A.); 3 Department of Pediatrics and Adolescent Medicine, Division of Pediatric Hematology and Oncology, Medical University of Graz, 8036 Graz, Austria; martin.benesch@klinikum-graz.at (M.B.); christian.urban@klinikum-graz.at (C.U.); marlene.mayer@klinikum-graz.at (M.M.); 4 Med Campus IV, University Clinic for Pediatrics, Kepler University Hospital, 4020 Linz, Austria; georg.ebetsbergerdachs@keplerklinikum.at; 5 Department of Pediatrics, Paracelsus Medical University, 5020 Salzburg, Austria; n.jones@salk.at (N.J.); a.gamper@salk.at (A.G.); 6 Department of Pediatric Hematology, Oncology, and Stem-Cell Transplantation, Medical University of Innsbruck, 6020 Innsbruck, Austria; bernhard.meister@i-med.ac.at (B.M.); roman.crazzolara@i-med.ac.at (R.C.); 7 Department of Pathology, Medical University of Vienna, 1090 Vienna, Austria; gabriele.amann@meduniwien.ac.at; 8 Department of Radiotherapy, Medical University of Vienna, 1090 Vienna, Austria; karin.dieckmann@meduniwien.ac.at; 9 Department of Pediatric Surgery, Medical University of Vienna, 1090 Vienna, Austria; ernst.horcher@meduniwien.ac.at; 10 Department of Pediatric Medicine, Landeskrankenhaus Leoben, 8700 Leoben, Austria; reinhold.kerbl@lkh-leoben.at; 11 Department of Pediatric Medicine, Medical University of Vienna, 1090 Vienna, Austria

**Keywords:** neuroblastoma, Austrian trial A-NB94, biomarkers

## Abstract

**Simple Summary:**

Neuroblastoma, the most common extracranial malignancy of childhood, shows a highly variable course of disease ranging from spontaneous regression or maturation into a benign tumor to an aggressive and intractable cancer in up to 60% of patients. To adapt treatment intensity, risk staging at diagnosis is of utmost importance. The A-NB94 trial was the first in Austria to stratify therapy intensity according to tumor staging, patient’s age, and *MYCN* amplification status, the latter being a biologic marker turning otherwise low-risk tumors into high-risk disease. Recent publications showed a prognostic impact of various genomic features including segmental chromosomal aberrations (SCAs). We retrospectively investigated the relevance of SCAs within this risk-adapted treatment strategy. The A-NB94 approach resulted in an excellent long-term survival for the majority of patients with acceptable long-term morbidity. An age- and stage-dependent frequency of SCAs was confirmed and SCAs should always be considered in future treatment decision making processes.

**Abstract:**

We evaluated long-term outcome and genomic profiles in the Austrian Neuroblastoma Trial A-NB94 which applied a risk-adapted strategy of treatment (RAST) using stage, age and *MYCN* amplification (MNA) status for stratification. RAST ranged from surgery only to intensity-adjusted chemotherapy, single or multiple courses of high-dose chemotherapy (HDT) followed by autologous stem cell rescue depending on response to induction chemotherapy, and irradiation to the primary tumor site. Segmental chromosomal alterations (SCAs) were investigated retrospectively using multi- and pan-genomic techniques. The A-NB94 trial enrolled 163 patients. Patients with localized disease had an excellent ten-year (10y) event free survival (EFS) and overall survival (OS) of 99 ± 1% and 93 ± 2% whilst it was 80 ± 13% and 90 ± 9% for infants with stage 4S and for infants with stage 4 non-MNA disease both 83 ± 15%. Stage 4 patients either >12 months or ≤12 months but with MNA had a 10y-EFS and OS of 45 ± 8% and 47 ± 8%, respectively. SCAs were present in increasing frequencies according to stage and age: in 29% of localized tumors but in 92% of stage 4 tumors (*p* < 0.001), and in 39% of patients ≤ 12 months but in 63% of patients > 12 months (*p* < 0.001). RAST successfully reduced chemotherapy exposure in low- and intermediate-risk patients with excellent long-term results while the outcome of high-risk disease met contemporary trials.

## 1. Introduction

Neuroblastoma is the most common extracranial malignancy of childhood and originates from the sympathetic nervous system. It shows a highly heterogeneous behavior ranging from spontaneous regression or maturation into a benign ganglioneuroma to an aggressive and intractable disease. Risk classification systems are using clinical and biological characteristics to predict survival and adapt treatment intensity [1,2].

At this study’s initiation, recognized risk driving factors included stage defined by the International Neuroblastoma Staging System (INSS), age at diagnosis, and *MYCN* oncogene amplification (MNA) status [3]. MNA, a strong biologic marker associated with rapid tumor growth [4,5], transforms otherwise favorable risk profiles of infants [6,7] and children with localized resectable [8,9] or unresectable [10] disease into high-risk [11]. Metastatic disease in children older than 18 months constitutes per se an unfavorable risk group regardless of *MYCN* status [12]. Intratumoral heterogeneous MNA (hetMNA) refers to the coexistence of clustered or scattered single MNA cells and non-*MYCN*-amplified (non-MNA) tumor cells [13], a phenomenon that was largely unexplored at the initiation of A-NB94. A recent study highlights the importance of viewing it separately from the MNA profile and its unfavorable risk implication, however, prognostication and therapy allocation are still unsolved issues [14,15].

Here, we present long-term outcomes of the Austrian neuroblastoma trial A-NB94, initiated in 1994 to apply a risk-adapted strategy of treatment (RAST) based on age (≤/>12 months), INSS stage and *MYCN* Status (Table 1). Encouraging results of the Lyon-Marseille-Curie-Est cooperative group (LMCE2) [16] using tandem high-dose chemotherapy (HDT) followed by autologous stem cell rescue (ASCR) prompted the adoption of a similar approach for patients with incomplete response to induction therapy. In addition, we show a post hoc genomic analysis to investigate pattern and potential influence of biomarkers on long-term outcomes.

## 2. Results

### 2.1. Trial Population and Overall Outcome

Between June 1994 and March 2006, a total of 163 patients were enrolled to the A-NB94 trial (Table 2) with most patients (*n* = 153) treated in five major Austrian centers. Histopathology revealed ganglioneuroblastoma (GNB) in 24/109 localized disease patients. Primary tumor locations were retroperitoneal-adrenal (*n* = 126; 77%), thoracic (*n* = 28; 17%), lumbar-pelvic (*n* = 5; 3%), and cervical (*n* = 4; 3%). The median age at diagnosis was 17 months (range: 4 days to 20 years); 63 patients were ≤12 months (39%), 22 patients between 12-18 months (13%) and 78 patients ≥ 18 months (48%). There were 86 males and 77 females. The 10-year (10y) EFS and OS were 80 ± 3% and 85 ± 3% for the whole trial population. The median observation time was twelve years.

### 2.2. Influence of Stage

**Figure 1:**
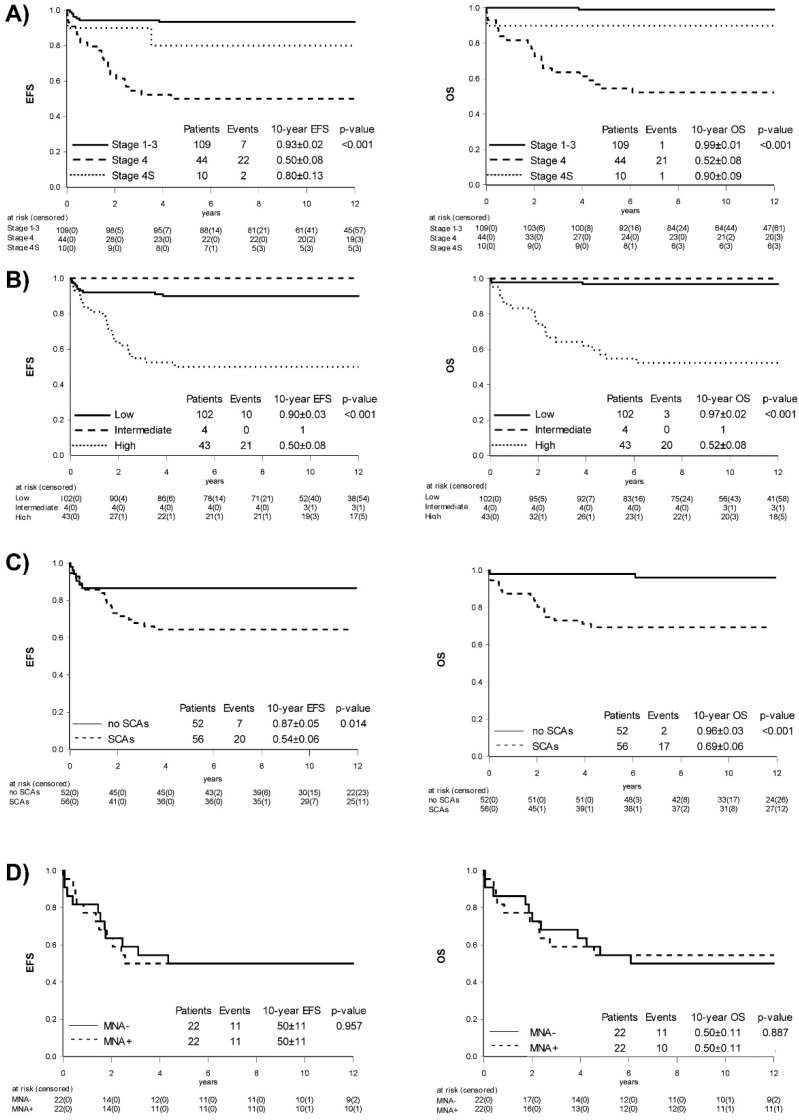
EFS and OS by (**A**) prospective International Neuroblastoma Staging System (INSS) as outlined in the A-NB94 trial; (**B**) post hoc grouping according to the International Neuroblastoma Risk Group (INRG) Staging System; (**C**) presence or absence of segmental chromosomal aberrations (SCAs); (**D**) effect of *MYCN* amplification (MNA) status in INSS stage 4 patients only.

Patients presenting with localized (stage 1–3) neuroblastoma (*n* = 109) had a 10y-EFS and OS of 93 ± 2% and 99 ± 1% (Figure 1A), respectively. Six relapses were reported and all affected non-MNA patients ≤ 12 months of age (*n* = 39). Four of these patients had loco-regional relapses: two were salvaged by six cycles cyclophosphamide/vincristine (CV), one by second surgery, and one was only observed as the parents declined further chemotherapy and the tumor ultimately regressed. Two infants had a relapse in infant age developing liver metastases, very much in line with a stage 4S pattern. They revealed no adverse genomic features at relapse, were closely observed, and ultimately showed spontaneous regression without further therapy. GNB was only found in localized non-MNA tumors and included the nodular (*n* = 4) or intermixed (*n* = 20) subtype. All patients with localized disease became long-term survivors, apart from one GNB patient with underlying neurofibromatosis type 1 dying later outside and unrelated to the A-NB94 trial, resulting in a 10y-EFS of 93 ± 3% for localized neuroblastoma versus 96 ± 4% for GNB (*p* = 0.584) with a 10y-OS of 100% versus 96 ± 4% (*p* = 0.062), respectively.

Infants with stage 4S neuroblastoma (*n* = 10) had a 10y-EFS and OS of 80 ± 13% and 90 ± 9% (Figure 1A), respectively. The tumors of four patients regressed without intervention while four patients underwent surgery of the primary tumor. Chemotherapy was given to two infants with clinical symptoms: one received electively four cycles of vincristine monotherapy and surgery whereas the other one, suffering from congenital neuroblastoma, showed uncontrollable disease progression, and died of respiratory failure despite chemotherapy escalation.

The 10y-EFS and OS of patients with metastasized neuroblastoma (*n* = 44) were 50 ± 8% and 52 ± 8% (Figure 1A), respectively. Six patients ≤ 12 months had non-MNA disease, a constellation associated with favorable outcome [6,7], but one patient died of progression during induction therapy. The subgroup at high risk for poor outcome including patients > 12 months with stage 4 (*n* = 33) and ≤ 12 months with MNA disease (*n* = 5) had a 10y-EFS and OS of only 45 ± 8% and 47 ± 8%.

In order to compare risk stratification of the A-NB94 trial with a more contemporary system, a retrospective assessment according to the International Neuroblastoma Risk Group (INRG) [2] was performed on this population. While patients with localized MNA tumors were upstaged to high-risk, localized, stage 4S, or stage 4 ≤ 12 months non-MNA patients remained low- or intermediate-risk, and stage 4 > 12 months and/or MNA patients remained in a high-risk group (Table A1). Outcome analysis resulted in 10y-EFS of 90 ± 3% for low- (*n* = 102), 100% for intermediate (*n* = 4), and 50 ± 8% for the high-risk group (*n* = 43) (*p* < 0.001) while 10y-OS was 97 ± 2%, 100%, and 52 ± 8% (*p* < 0.001) (Figure 1B), respectively.

Anti-GD2 monoclonal antibody ch14.18/SP0/2 became available in 1996 for compassionate use in 12 stage 4 patients. The landmark time identified the median time between HDT and initiation of immunotherapy as 87 days. When comparing the anti-GD2 monoclonal antibody pilot population (*n* = 12) to the pre-immunotherapy population accrued in the A-NB94 trial and considering only stage 4 patients without progressive disease at the landmark time point of 87 days after HDT, no difference in outcome was observed shown by a 10y-EFS of 67 ± 14% versus 64 ± 13% (*p* = 0.77) and a 10y-OS of 67 ± 14% versus 71 ± 12% (*p* = 0.907).

### 2.3. Role of Age

Using a cutoff at 12 months as part of RAST, 10y-OS was significantly better with 94 ± 3% for patients ≤ 12 months (*n* = 63) as compared to 80 ± 4% for patients > 12 months (*n* = 100) (*p* = 0.035); EFS was 83 ± 5% and 79 ± 4% (*p* = 0.717), respectively. An ad hoc cutoff at 18 months showed similar results with a 10y-OS of 93 ± 3% for patients ≤ 18 months (*n* = 85) versus 77 ± 5% for patients > 18 months (*n* = 78) (*p* = 0.009) and a 10y-EFS of 85 ± 4% versus 75 ± 5% (*p* = 0.201).

### 2.4. Occurrence and Influence of Biomarkers 

*MYCN* status was evaluated for all patients prospectively during the risk stratification process. *MNA* was observed in 6% (7/109) of localized, in 43% (20/44) of stage 4, and in none of the stage 4S tumors (*p* < 0.001). We found four infants harboring het*MNA*; one of them was only recognized in a later tumor sample and this patient was treated according to the original result as per *MNA* protocol. The other three patients were treated in the non-*MNA* arm.

*MYCN* retained predictive power in the total trial population showing a 10y-EFS of 84 ± 3% for non-*MNA* (*n* = 133), 60 ± 10% for *MNA* (*n* = 26), and 100% for het*MNA* (*n* = 4) (*p* = 0.034), and a 10y-OS of 64 ± 10%, 89 ± 3%, and 100% (*p* = 0.008), respectively. However, *MYCN* amplification did not have any additional stratifying effect on outcome in patients with stage 4 neuroblastoma as 10y-EFS and OS was 50 ± 11% for both subgroups (Figure 1D).

1p^loss^ was recorded prospectively during the active trial period and data were available for 122 patients. Quantity and quality of frozen tumor samples allowed for post hoc genomic analysis of 108 patients (multiplex ligation-dependent probe amplification (MLPA), *n* = 68; single nucleotide polymorphism (SNP) array, *n* = 32; interphase fluorescent in-situ hybridization (iFISH), *n* = 8) including SCAs for 1q^gain/1qloss^, 2p^gain^, 3p^loss^, 4p^loss^, 5p^loss^, 6q^loss^, 9p^loss^, 11q^loss^, 14q^loss^, 17p^loss^, 17q^gain^, 19q^loss^, and 22q^loss^. Only in two cases of GNB, genomic analysis was interpretable and revealed no SCAs (data included in mentioned numbers); in the other Schwann cell stroma-rich GNB tumors, the neoplastic clone was masked by the normal Schwann cells [17].

**Figure 2:**
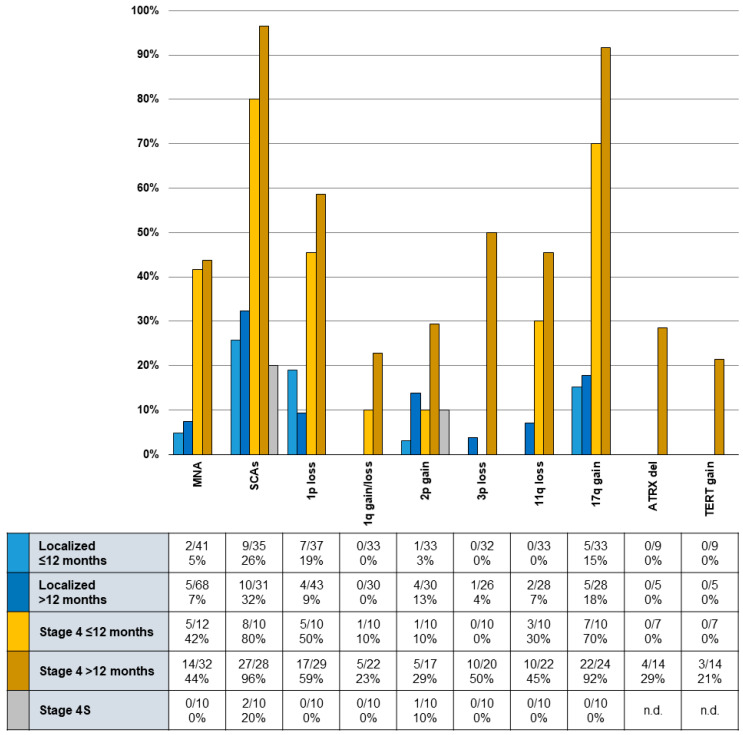
Presence of SCAs, *ATRX* deletion, and *TERT* gain within groups by age and stage. Presence of segmental chromosomal alterations (SCAs) that were associated with a significantly lower event-free (EFS) and overall survival (OS) in a univariate analysis within groups differentiated by age (≤/>12 months) and stage by International Neuroblastoma Staging System (INSS) (localized/4S or stage 4).

SCAs were observed in 92% of stage 4 but only in 29% of localized tumors (*p* < 0.001). Patients > 12 months showed tumor SCAs in 63% while SCAs were found in only 39% of patients ≤ 12 months (*p* < 0.001) (Figure 2).

This analysis found patients with tumors showing one or more SCAs (*n* = 56) with a 10y-EFS and OS of 64 ± 6% and 69 ± 6% while it was 87 ± 5% (*p* = 0.014) and 96 ± 3% (*p* < 0.001) for patients without SCAs (*n* = 52) (Figure 1C). In univariate analysis, presence of 1p^loss^, 1q^gain^/1q^loss^, 3p^loss^, 11q^loss^, or 17q^gain^ had significant predictive power for worse 10y-EFS and/or OS (Table 3). To investigate the potential added impact of segmental chromosomal alterations (SCAs), we performed a multivariate analysis (MVA) corrected by the risk-stratifying factors of age (≤/>12 months), INSS stage and *MYCN* amplification status. Neither model A nor model B (Table 4) was able to identify an added risk for SCAs in the investigated trial population. *ATRX*^del^ (*n* = 3) and *TERT*^gain^ (*n* = 4) were found only in stage 4 patients > 18 months of age and were mutually exclusive. All but one patient with *TERT*^gain^ died with progressing disease.

### 2.5. Role of Treatment Elements to Achieve Remission Induction

Low- to intermediate-dose chemotherapy during first-line treatment was given to 18% (20/109) of patients with localized disease, 20% (2/10) of patients with stage 4S, and 100% (6/6) of patients ≤ 12 months with stage 4 non-MNA tumors. The overall response rate to cytotoxic treatment was 93% (26/28) in this cohort with 50% (14/28) entering a complete clinical remission (CR). One patient with stage 4 non-MNA and one with stage 4S disease did not respond, and both died of disease progression despite chemotherapy escalation.

Stage 4 patients > 12 months (*n* = 32) and ≤12 months with MNA tumors (*n* = 6) received dose-intensive induction therapy with a metastatic response rate of 74% (28/38) and a CR rate of 16% (6/38) including the effects of surgery. In this group, three patients progressed during induction. 2/38 patients (5%) experienced an infection-related septic shock during induction therapy and died with multi-organ failure.

#### 2.5.1. Surgery

Surgical resection of the primary tumor was performed in 93% (152/163) of patients. In case of adrenal primary, 5% (6/113) underwent unilateral total nephrectomy while other patients only had unilateral adrenalectomy. Two patients needed revision surgery due to rebleeding.

#### 2.5.2. High-Dose Therapy (HDT)

Patients eligible for HDT (Table 1) received one (*n* = 18), or, in case of mIBG metastatic incomplete response, two (*n* = 11) or three (*n* = 2) courses of HDT. In addition, two patients with stage 3 *MNA* but post-surgical macroscopic tumor residues, and one patient 11 months of age with stage 4 non-*MNA* but post-induction unresectable disease received a single course of HDT. Seven patients with single HDT were in metastatic CR (mCR) prior HDT; 93% (13/14) of other patients responded to HDT with a mCR rate of 50% (7/14). Within the multiple HDT group, response rate was 77% (10/13), CR rate 54% (7/13). 

In the first attempt of stem cell apheresis in 27 evaluable patients, median yield was 4.5 × 10^6^ CD34 positive cells per kilogram body weight (range 0.45 to 30.9 × 10^6^). Six patients needed a second (median yield 3.76 × 10^6^, range 1.26 to 8.5 × 10^6^), and two patients a third apheresis (median 4.99 × 10^6^, range 4.97 to 5 × 10^6^) in order to reach the attempted minimum of 3 × 10^6^ cells per kilogram body weight for each of the planned HDT courses according to their respective response status. 

### 2.6. Acute Toxicities and Long-Term Morbidity

Acute toxicities and intervention related morbidity was related to respective treatments (Table A2) and clearly showed a higher acute toxicity burden with increased treatment intensity. 

Of surviving patients receiving low- and intermediate-dose chemotherapy (localized, stage 4S, or stage 4 patients ≤12 months with non-MNA disease) but no HDT (*n* = 24), only two patients had treatment-associated long-term disabilities manifesting as reduced renal glomerular filtration rate (GFR). Treatment-unrelated morbidity was persistent paraplegia of the lower limbs already present at diagnosis (*n* = 1) and subsequent craniopharyngioma (*n* = 1).

Of long-term survivors receiving intensive chemotherapy including HDT (*n* = 21), common long-term morbidity involved permanent hearing loss after cisplatin therapy in 43% (*n* = 9). Other disabilities in this group included reduced left-ventricular cardiac output (*n* = 4), hypothyroidism (*n* = 3), reduced GFR (*n* = 2), hypertension (*n* = 1), testosterone deficiency (*n* = 1), growth hormone deficiency (*n* = 1), dental damage (*n* = 1), peripheral polyneuropathy (*n* = 1), and osteochondroma (*n* = 1). One patient died from secondary acute myeloid leukemia.

## 3. Discussion

The A-NB94 trial was the first in Austria to apply a risk-adapted treatment strategy for children with neuroblastoma considering INSS stage, age, and *MYCN* amplification status [3]. Risk stratification was implemented in 1994 and results need to be viewed from this perspective. Only 15 years later, the INRG established staging based on pre-surgical image-defined factors [18], and included additional biologic makers of histopathology, ploidy, and 11q status [2]. We retrospectively stratified A-NB94 patients according to the INRG classification system which resulted in an upstaging of patients with localized MNA tumors to high-risk while other patients remained within a risk group similar to the A-NB94 staging system based on the INSS. Additionally, outcome by INRG classification matched published results, considering the high survival of stage 3 patients and small numbers within the intermediate-risk group. Patients being not stageable by INRG classification related to missing 11q^loss^ data.

In retrospect, the A-NB94 study included a high proportion of patients with localized neuroblastoma, possibly related to the then ongoing neuroblastoma screening with 13% of the trial population being part of this program. In addition, the high-risk arm of A-NB94 closed for accrual earlier to allow for participation in the SIOPEN High-Risk trial (HR-NBL1/SIOPEN; ClinicalTrials.gov number NCT01704716) which opened already in 2002 while the SIOPEN Low- and Intermediate-Risk trial (LINES; ClinicalTrials.gov number NCT01728155) only opened in 2007.

Compared with published data, the Austrian screening program detected a comparatively high number of localized tumors harboring unfavorable biologic features (MNA, 1p^loss^, diploidy) [19], suggesting early adverse clonal evolution. However, the A-NB94 trial was not designed for answering the question if screening helped increasing the detection of high-risk disease at an early stage [20,21]. Overall, our approach resulted in excellent survival for these patients as all became long-term survivors apart from one GNB patient dying from complications after surgery not related to neuroblastoma treatment.

Notably, the very favorable outcome of the localized subgroup also includes 25 stage 3 patients, marking a major improvement compared to the preceding A-NB87 trial which applied an intensive chemotherapy for children with Evan’s Stage 3 disease (especially when >2 years of age, increased neuron-specific enolase, and/or ferritin (Table A3), resulting in a 28% (8/29) toxic death rate and a 5y-OS of only 50% [22]. However, detailed comparison is hampered by differing staging criteria and *MYCN* not being a stratifying marker in A-NB87. In A-NB94, 82% (89/109) of patients with localized neuroblastoma received little or no chemotherapy in first line treatment. These numbers include six patients with non-*MNA* stage 3 tumors defined by tumors crossing the midline which could otherwise be surgically removed upfront. While two relapses were treated successfully with chemotherapy, potential long-term side effects following intense therapy could be circumvented in other patients by using a surgery only approach. 

While our data support the notion of improved outcome despite therapy reduction in patients with intermediate-risk neuroblastoma [23,24], contemporary trials would submit patients with stage 2 or 3 MNA [25] or diploid [26] tumors to receive HDT/ASCR. Furthermore, the presence of certain SCAs was reported to identify patients of higher risk for relapse, especially in unresectable tumors [27]. While SCAs, especially 1p^loss^ and 11q^loss^, may lower EFS but not OS in children ≤18 months, they also diminish OS in older children [28]. In A-NB94, 15 patients with localized tumors matched these criteria (MNA, *n* = 7; diploidy, *n* = 5; >18 months and 1p^loss^, *n* = 1; or 11q^loss^, *n* = 2) and all survived without relapse after receiving low-/intermediate-dose chemotherapy apart from two already described patients that received HDT/ASCR. The decision for adding HDT in the latter two patients was based on the presence of post-surgical residual tumors. These small numbers might suggest that patients with residual tumors benefited from therapy escalation while others were adequately treated by conventional chemotherapy and irradiation. In this context, it may be assumed that the remarkable good outcome in A-NB94 of patients with localized tumors including especially stage 3 patients relates to the surprisingly low prevalence of SCAs observed in this subgroup.

Outcome of infantile stage 4 metastatic disease is related to *MNA*, diploidy/tetraploidy, 1p^loss^, 11q^loss^, or 17q^gain^ [29]. While most stage 4S tumors are treated adequately by observation only [30,31], certain markers including 11q^loss^ and diploidy might predict less favorable outcomes even in stage 4S disease [32]. However, disease progression of two A-NB94 stage 4S patients clearly could not be explained by these biologic factors, as neither *MNA,* diploidy or 11q^loss^ were detected. Stage 4S poses threats independent of tumor biology as extensive hepatomegaly causes a variety of clinical complications.

Of 44 stage 4 patients, six infants without *MNA* received moderate chemotherapy without HDT or radiation according to RAST. While survival was good, one of three patients with tumors showing SCAs involving 11q^loss^ and 17q^gain^ progressed and died during induction, in line with reports that these features are often associated with higher risk for relapse [33].

Stage 4 patients > 12 months and stage 4 infants with *MNA* [34,35] underwent an induction protocol similar to the “N6” protocol previously published to achieve fast cytoreduction [36], following the notion that increased response rates precede higher survival rates [37]. Furthermore, A-NB94 successfully introduced tandem or triple HDT/ASCR to children with incomplete metastatic response to induction therapy. While benefits of multiple cycle HDT have been reported consistently by European groups [16,38], recent reports demonstrated clearly improved survival in contemporary trials [39,40].

In 1996, anti-GD2 monoclonal antibody ch14.18/SP0/2 became available for compassionate use in stage 4 patients. The identified landmark time identified between HDT and start of immunotherapy was 87 days; thus, only stage 4 patients without progressive disease were included in the pre-immunotherapy control population. Comparing these two cohorts did not result in an advantage for the small immunotherapy population. However, overall outcome data are quite in line with later publications on the use of ch14.18 monoclonal antibody in first-line maintenance treatment [41,42]. Considering the rather small population in both cohorts, we only may hypothesize that RAST, using repetitive HDT adapted to response in this high-risk population, was probably a major contributor to the observed favorable outcomes and that immunotherapy applied with a less dose intensive monotherapy schedule did not result in a measurable added benefit.

In addition, while the prognostic impact of het*MNA* remains unclear [43,44], recent data suggests it has to be viewed in light of the overall genetic background in order to determine the importance of the *MNA* cell clone [45]. Localized tumors usually show a favorable background with reported improved outcomes compared to homogeneous *MNA*. In A-NB94, het*MNA* tumors showed only additional 1p^loss^ in two cases but no other SCA or other unfavorable biologic markers. One of these cases was only discovered in a later tumor sample as the original sample suggested homogeneous *MNA*; this patient with stage 4 disease received high-dose therapy, which may, together with the age of ≤12 months and lack of additional high-risk features, have resulted in over-treatment.

SCAs were present in higher frequency in stage 4 disease, especially in patients > 12 months, when compared to children with localized tumors of any age (Figure 2), supporting the notion of SCAs being accumulated during tumor development [44] and an unfavorable biology being associated with overall poor prognosis [45]. This study confirms SCAs are commonly involving chromosome arms 1p, 11q, and 17q [46], however, high-risk metastasized tumors often show a combination of various chromosomal defects [47].

The acute toxicity burden as well as long-term morbidity is clearly related to RAST, showing the therapy burden of the curative efforts mainly in the high-intensity treatment groups. Particularly after additional HDT/ASCR, which is in line with previous reports [48,49], although and in contrast to previous approaches [50], no total body irradiation (TBI) was used. Observed surgery-related morbidities were in line with previous reports [51]. Irreversible inner ear hearing loss, a known side-effect following cisplatin therapy [52], affected 29% (10/34) of long-term survivors receiving cisplatin. The understanding of the mechanism of uptake and accumulation in the stria vascularis of the cochlea provides now an important target for preventing ototoxicity in future trials [53]. Overall, organ-related long-term side-affects were low as well as incidence of secondary malignancies.

## 4. Patients and Methods

### 4.1. Patients

Children with histologically confirmed, previously untreated neuroblastoma or GNB up to 20 years of age were eligible for enrolment on A-NB94. Staging followed INSS guidelines and treatment response was assessed by the 1993 International Neuroblastoma Response Criteria (INRC) [3]. Written informed consent for treatment and data procession was obtained in compliance with institutional review board rules and in accordance with the Declaration of Helsinki [54].

### 4.2. Treatment Concepts

RAST was adjusted for age (≤/>12 months), stage, and *MYCN* status (Table 1). Stage 1 or 2 non-*MNA* tumors were planned for surgery only; incompletely resected *MNA* tumors received chemotherapy adapted to stage and radiotherapy. Stage 4S infants were observed up to 3 months and then underwent surgery but received chemotherapy in case of life-threatening symptoms or progression. Stage 3 and 4 were treated with neoadjuvant intensity-adjusted chemotherapy with surgery planned after four cycles. *MNA* tumors were irradiated age-adapted with 24 Gray (≤12 months) or 30 Gray (>12 months).

Stem cell harvest, aiming at >3 × 10^6^ CD34 positive cells per kilogram body weight per high-dose treatment (HDT), was first attempted after 4 chemotherapy cycles if cytomorphological and histological bone marrow (BM) remission was achieved, or was otherwise postponed following cycle 6 or after an in vivo purge following the first or second HDT, eventually.

Depending on metastatic response, patients were eligible for a single (complete response, CR) or repetitive HDT (partial/minor response, PR/MR). In case of CR after second HDT, the third HDT was omitted. In 1996, the anti-GD2 monoclonal antibody ch14.18/SP0/2 became accessible as compassionate use (provided by the University of Tübingen, Prof. Dr. Rupert Handgretinger) to optimize treatment of HR disease after HDT and radiotherapy. Treatment consisted of three anti-GD_2_ ch14.18/SP0/2 cycles (20 mg/m^2^ over 5 days as 8-h infusions). Supportive care and treatment of infections followed institutional guidelines.

### 4.3. Disease and Response Assessment

Disease evaluation was planned at diagnosis, after 2, 4, and 6 courses of chemotherapy, before and after each HDT, and before and after immunotherapy. Bone marrow examination included aspirates/trephines obtained from two sites and used, apart from cytomorphology, an automated image analysis system (MetaSystems GmbH, Altlußheim, Germany) to quantify GD_2_/CD56-positive cells [55,56]. Skeletal disease was defined by pathologic iodine-123 *meta*-iodobenzylguanidine (mIBG) or, in mIBG non-avid tumors, by tecnetium-99m-hydroxymethylenediphosphanate (Tc99m) scans [57]. Primary tumors were investigated by MRI/CT scan. Tumor marker evaluation included urinary catecholamine metabolites, lactate dehydrogenase (LDH), and neuron specific enolase (NSE). Response was assessed based on local institution reporting following INRC guidelines [3].

### 4.4. Toxicity Evaluation

Acute and long-term toxicities were evaluated using case report forms (CRF) documenting organ-specific toxicities according to the Common Terminology Criteria for Adverse Events (CTCAE) Version 5.0 above grade 2, secondary malignancies, and other adverse events (AE). Acute toxicity evaluation focused on patients with intense induction chemotherapy and HDT.

### 4.5. Biologic Studies

Frozen tumor specimens were evaluated using high-density SNP arrays (CytoScan HD Array) [47], MLPA, or iFISH for the detection of *MNA* or SCAs. Recording/interpretation of data were done according to international standards [58].

### 4.6. Statistical Analysis

Event-free survival (EFS) was calculated from the time of diagnosis until first occurrence of relapse, progressive disease, secondary malignancy, or death from any cause, or until last contact with patients. Overall survival (OS) was calculated from time of diagnosis to death from any cause. The median time between HDT and initiation of immunotherapy was 87 days, therefore only patients without progressive disease at this landmark time point were included in the pre-immunotherapy cohort. Categorical data were analyzed by chi-square test or, in case of an estimated case-count of ≤5 per field, Fisher’s exact test. Data were analyzed with SAS 9.4 program. All statistical tests were two-sided.

## 5. Conclusions

The risk-adapted approach resulted in an excellent long-term survival for the majority of patients with acceptable long-term morbidity. An age- and stage-dependent frequency of SCAs was confirmed and should be considered in future treatment decision-making processes.

## Figures and Tables

**Table 1:** Overview of the A-NB94 risk-adapted strategy of treatment.

**Risk-Adapted Strategy of Treatments in the A-NB94 Trial**								
**Stage**		**Age**	***MYCN*** **Non-Amplified**			***MYCN*** **Amplified**		
1, 2		≤12	surgery			surgery if microscopic incomplete resection: 6 × CAV, radiotherapy		
		>12				surgery if microscopic incomplete resection: 3 × alternating CAV + CBDCA/VP16, radiotherapy		
3		≤12	4–6 × CV surgery			3 × alternating CAV + CBDCA/VP16 surgery radiotherapy		
		>12	3 × alternating CAV + CBDCA/VP16 surgery			3 × alternating HD-CAV + CDDP/VP16 surgery radiotherapy		
4		≤12				3 × alternating HD-CAV + CDDP/VP16 surgery HDT/ASCR (single or multiple) radiotherapy		
		>12	3 × alternating HD-CAV + CDDP/VP16 surgery HDT/ASCR (single or multiple) radiotherapy					
4S		≤12	observation (up to 3 months) if progression: surgery if life-threatening symptoms: CV option to escalation: CBDCA/VP16			-		
**Details on Chemotherapy**								
**Chemotherapy**				**Abbreviation**	**Substance**		**Dosage**	**Days Given**
CV				CYC	cyclophosphamide		5 mg/kg	1–5
				VCR	vincristine		0.05 mg/kg	1
CAV				CYC	cyclophosphamide		300 mg/m^2^	1–5
				ADR	doxorubicin		60 mg/m^2^	5
				VCR	vincristine		1.5 mg/m^2^	1, 5
CBDCA/VP16				CBDCA	carboplatin		200 mg/m^2^	1–3
				VP16	etoposide		150 mg/m^2^	1–3
HD-CAV				CCY	cyclophosphamide		70 mg/kg	1, 2
				ADR	doxorubicin		25 mg/m^2^	1–3
				VCR	vincristine		1 (1.5) mg/m^2^	1–3 (9)
CDDP/VP16				CDDP	cisplatin		40 mg/m^2^	1–5
				VP16	etoposide		150 mg/m^2^	3–5
single HDT				VP16	etoposide		60 mg/kg	−4
				CBDCA	carboplatin		500 mg/m^2^	−4–2
				MEL	melphalan		180 mg/m^2^	−2
multiple HDT	1st course			THIO	thiotepa		200 mg/m^2^	−5–3
				CBDCA	carboplatin		500 mg/m^2^	−5–3
	2nd course			THIO	thiotepa		200 mg/m^2^	−5–3
				CYC	cyclophosphamide		1500 mg/m^2^	−4–2
	3rd course			VP16	etoposide		40 mg/kg	−3
				MEL	melphalan		140 mg/m^2^	−2

Therapy intensity was adapted by the risk-stratifying factors of age (≤/>12 months), disease stage according to the International Neuroblastoma Staging System (INSS), and *MYCN* amplification (MNA) status. In treatment arms including neoadjuvant chemotherapy, surgery was attempted after 4 cycles. Radiotherapy to the primary tumor was 24 Gray (Gy) for patients ≤ 12 months and 30 Gy for patients ≥ 12 months. Dosage for infants was calculated according to their bodyweight instead of body surface area.

**Table 2:** Characteristics of the A-NB94 study cohort.

		Total				Non-MNA			MNA			hetMNA		
Age		<12	≥12	Total	%	<12	≥12	Total	<12	≥12	Total	<12	≥12	Total
INSS Stage	GNB	2	22	24	15%	2	22	24						
	1	16	30	46	28%	14	29	43	1	1	2	1		1
	2	9	7	16	10%	8	7	15				1		1
	3	14	9	23	14%	12	5	17	1	4	5	1		1
	4	12	32	44	27%	6	18	24	5	14	19	1		1
	4S	10		10	6%	10		10						
	Total	63	100	163	100%	52	81	133	7	19	26	4		4
	**%**	39%	61%	100%		32%	50%	82%	4%	12%	16%	2%		2%

Characteristics of the A-NB94 study cohort including risk-stratifying factors of disease stage according to the International Neuroblastoma Staging System (INSS), age (≤ or >12 months), and *MYCN* oncogene amplification (MNA) status including heterogeneous MNA (hetMNA). To appreciate differences in histopathology, we separated ganglioneuroblastoma (GNB) from other localized disease.

**Table 3:** Summary of 10-year outcomes with reference to biologic markers.

Biologic Criterion			EFS			OS		
Marker		*n*	Events	10y	*p*	Deaths	10y	*p*
*MYCN*	normal	133	21	84 ± 3	0.035	14	89 ± 3	0.008
	MNA	26	10	60 ± 10		9	64 ± 10	
	hetMNA	4	0	100		0	100	
SCAs	absent	52	7	87 ± 5	0.014	2	96 ± 3	<0.001
	present	56	20	64 ± 6		17	69 ± 6	
1p	normal	89	14	84 ± 4	0.015	10	89 ± 3	0.004
	loss	33	12	64 ± 8		10	69 ± 8	
1q	normal	91	18	81 ± 4	<0.001	10	89 ± 3	<0.001
	gain/loss	6	6	0		6	0	
2p	normal	80	18	78 ± 5	0.702	11	86 ± 5	0.562
	gain	12	2	83 ± 11		2	83 ± 11	
3p	normal	83	18	78 ± 5	0.007	11	87 ± 4	0.002
	loss	10	6	40 ± 15		5	50 ± 16	
4p	normal	87	21	76 ± 5	0.512	14	87 ± 5	0.119
	loss	7	1	86 ± 13		0	100	
11q	normal	80	15	81 ± 4	0.002	8	90 ± 3	<0.001
	loss	17	9	47 ± 12		9	47 ± 12	
14q	normal	70	17	75 ± 5	0.971	12	83 ± 5	0.982
	loss	12	3	75 ± 13		2	83 ± 15	
17q	normal	58	9	84 ± 5	0.006	3	95 ± 3	<0.001
	gain	38	16	58 ± 8		15	61 ± 8	

10-year (10y) event-free survival (EFS) and overall survival (OS) according to the univariate analysis of biologic markers including *MYCN* amplification (MNA) and heterogeneous MNA (hetMNA), and presence or absence of segmental chromosomal alterations (SCAs).

**Table 4:** Multivariate model of SCAs corrected by stage, age, and MNA.

Model	Marker	EFS			OS		
		HR	95% CL	*p*	HR	95% CL	*p*
A	SCAs	0.67	0.15−2.91	0.59	1.06	0.16−6.93	0.95
B	1 p^loss^	0.83	0.19−3.63	0.80	0.38	0.07−1.97	0.25
	1 q^gain/loss^	1.68	0.43−6.53	0.46	2.45	0.56−10.7	0.23
	3 p^loss^	0.95	0.26−3.52	0.94	0.62	0.15−2.66	0.52
	11 q^loss^	1.69	0.46−6.23	0.43	2.08	0.50−8.74	0.32
	17 q^gain^	0.30	0.03−3.52	0.34	2.59	0.26−26.2	0.42

Multivariate analysis (MVA) showing the hazard ratio (HR) with 95% confidence level (CL), event-free survival (EFS), and overall survival (OS) of segmental chromosomal alterations (SCAs). Model A showing MVA of all SCAs combined (including 1p^loss^, 1q^gain^/1q^loss^, 2p^gain^, 3p^loss^, 4p^loss^, 5p^loss^, 6q^loss^, 9p^loss^, 11q^loss^, 14q^loss^, 17p^loss^, 17q^gain^, 19q^loss^, and 22q^loss^). Model B showing MVA of specific SCAs univariately significant for a lower EFS and OS.

## Data Availability

Biomarker data as presented in this cohort study are available in anonymized form on request at the CCRI by addressing the corresponding author.

## Appendix A

**Table A1:** Retrospective correlation of A-NB94 risk groups with INRG stratification.

A-NB94 Staging Criteria			Retrospective INRG Risk Classification				
*MYCN*	Stage	Age	Low	Intermediate	High	n.e.	Total
amplified	1	≤12			1		1
		>12			1		1
	2	≤12					
		>12					
	3	≤12			1		1
		>12			4		4
	4	≤12			5		5
		>12			14		14
non-amplified	1	≤12	15				15
		>12	46				46
	2	≤12	9				9
		>12	10				10
	3	≤12	8			4	12
		>12	3	3		1	7
	4	≤12	4	1		1	6
		>12			17	1	18
	4S	≤12	4			6	10
heterogeneously amplified	1	≤12	1				1
		>12					
	2	≤12	1				1
		>12					
	3	≤12	1				1
		>12					
	4	≤12				1	1
		>12					
Total			102	4	43	14	163

Retrospective correlation of A-NB94 risk stratification with the International Neuroblastoma Risk Group (INRG) classification [2].

**Table A2:** Summary of acute treatment-related toxicities.

Type of Toxicity	Induction Phase		Surgery	HDT Phase
	LD/ID	HD		
infection	4	10	4	13
gastrointestinal		3		8
hepatic injury		3		
venous occlusive disease				1
cardiac	2	3		4
renal failure				2
hemorrhagic cystitis				1
respiratory insufficiency				1
oral-intestinal mucositis		5		12
tumor lysis syndrome		1		
capillary leak syndrome		1		1
hemolytic uremic syndrome				1
autoimmune hemolytic anemia				1
non-febrile seizures				1
intraoperative tumor rupture			2	
severe bleeding			4	
lymphatic fistula			4	
Horner’s syndrome			3	
pleural effusion			2	
pneumothorax			1	
cava vein thrombosis			1	
Total	6	26	21	46

Summary of acute treatment-related toxicities according to induction treatment split by chemotherapy intensity with low- and intermediate-dose (LD/ID) and high-dose (HD) induction schedules, the latter including stage 4 > 12 months and stage 4 ≤ 12 months but *MYCN* amplified tumors, the former all other treatment groups; surgical complications; and high-dose therapy (HDT).

**Table A3:** Chemotherapy elements and treatment regimens used in the A-NB87 trial.

Stage	Therapy								
**I**	Surgery								
**IIA**	Surgery	If macroscopic residual tumor: second surgery, or radiotherapy							
**IIB**	Surgery	CV			Re-surgery		Radiotherapy		
**IIIA**	Surgery	3–4 × alternating DAMO/MVDOC			Re-surgery		Radiotherapy		(Re-surgery)
**IIIB**	Surgery	3–5 × alternating DAMO/MVDOC/IPE			Re-surgery		Radiotherapy		Re-surgery
**IV**	Surgery	4 × alternating MVDOC/IPE			Re-surgery		HDT/TBI/ASCR		Radiotherapy
**Details on Chemotherapy**									
**Chemotherapy**			**Abbreviation**	**Substance**		**Dosage**		**Days Given**	
DAMO			D	dacarbazine		850 mg/m²		1	
			A	doxorubicin		30 mg/m²		1, 2	
			M	mustargen		6 mg/m²		1	
			O	vincristine		1.5 mg/m²		1 + 5	
MVDOC			M	mustargen		6 mg/m²		1	
			V	teniposide		150 mg/m²		1	
			D	dacarbacine		850 mg/m²		1	
			O	vincristine		1.5 mg/m²		1	
			C	cyclophosphamide		850 mg/m²		1	
IPE			I	ifosfamide		3 g/m²		1, 2	
			P	cisplatin		40 mg/m²		1–5	
			E	etoposide		150 mg/m²		3–5	

Chemotherapy elements and treatment regimens according to the Austrian Neuroblastoma Trial A-NB87. Staging was done according to Evans criteria: stage I (localized without macroscopic lymphatic node involvement), stage IIA (macroscopic lymphatic node involvement), IIB (macroscopic residual tumor with ipsilateral lymphatic node involvement), IIIA (<2 years of age, ferritin < 300 µg/mL, neuron-specific enolase < 100 ng/mL), IIIB (any positivity of the markers described for stage IIIA), stage 4 (distant metastasized disease).
